# Smac mimetic-derived augmentation of chemotherapeutic response in experimental pancreatic cancer

**DOI:** 10.1186/1471-2407-11-15

**Published:** 2011-01-12

**Authors:** Niranjan Awasthi, Amanda Kirane, Margaret A Schwarz, Jason E Toombs, Rolf A Brekken, Roderich E Schwarz

**Affiliations:** 1 Division of Surgical Oncology, Department of Surgery, The University of Texas Southwestern Medical Center Dallas, TX 75390, USA; 2 Department of Pediatrics, The University of Texas Southwestern Medical Center Dallas, TX 75390; 3 Hamon Center for Therapeutic Oncology Research, Simmons Comprehensive Cancer Center, The University of Texas Southwestern Medical Center Dallas, TX 75390; 4 Department of Pharmacology The University of Texas Southwestern Medical Center Dallas, TX 75390

## Abstract

**Background:**

Pancreatic ductal adenocarcinoma (PDAC) is highly resistant to conventional chemotherapy, in part due to the overexpression of inhibitors of apoptosis proteins (IAPs). Smac is an endogenous IAP-antagonist, which renders synthetic Smac mimetics attractive anticancer agents. We evaluated the benefits of combining a Smac mimetic, JP1201 (JP), with conventional chemotherapy agents used for PDAC management.

**Methods:**

Cell viability assays and protein expression analysis were performed using WST-1 reagent and Western blotting, respectively. Apoptosis was detected by annexin V/propidium iodide staining. *In vivo *tumor growth and survival studies were performed in murine PDAC xenografts.

**Results:**

JP and gemcitabine (Gem) inhibited PDAC cell proliferation with additive effects in combination. The percentage of early apoptotic cells in controls, JP, Gem and JP + Gem was 17%, 26%, 26% and 38%, respectively. JP-induced apoptosis was accompanied by PARP-1 cleavage. Similar additive anti-proliferative effects were seen for combinations of JP with doxorubicin (Dox) and docetaxel (DT). The JP + Gem combination caused a 30% decrease in tumor size *in vivo *compared to controls. Median animal survival was improved significantly in mice treated with JP + Gem (38 d) compared to controls (22 d), JP (28 d) or Gem (32 d) (p = 0.01). Animal survival was also improved with JP + DT treatment (32 d) compared to controls (16 d), JP (21 d) or DT alone (27 d).

**Conclusions:**

These results warrant further exploration of strategies that promote chemotherapy-induced apoptosis of tumors and highlight the potential of Smac mimetics in clinical PDAC therapy.

## Background

Pancreatic ductal adenocarcinoma (PDAC) is the fourth leading cause of cancer-related deaths in the United States. Prognosis of PDAC patients is very poor mostly due to the late diagnosis, aggressive nature of disease and an unusually high resistance to chemotherapy and radiation [1-3]. Despite advancements in diagnostic and surgical procedures and treatments, the overall 5-year survival remains less than 5% [1]. Surgical resection remains the only option for long-term survival of patients. However, locally extended and metastatic disease limits the use of this procedure to only about 10% of patients [4]. Therefore, the majority of pancreatic cancer patients are treated with systemic therapies. Gemcitabine (Gem), a fluorinated pyrimidine antagonist, is currently the most active single agent for locally advanced, non-operable and metastatic PDAC. However, Gem is only effective in a subset of patients, and improvements in overall survival remain considerably modest [5]. Several other cytotoxic and chemotherapy agents such as cisplatin, fluorouracil, erlotinib, oxaliplatin, docetaxel and irinotecan have been tested as second-line chemotherapy or in combination with Gem for PDAC. However, most of these studies have failed to show any significant improvement in overall patient survival compared to single agent Gem [6-12]. Therefore, there is an urgent need for the development of therapeutic strategies that target novel mechanisms, and are either effective alone or enhance the activity of standard agents.

Many cancer cells possess apoptotic dysfunction that correlates with tumor aggressiveness and resistance to conventional chemotherapy [13]. Various antiapoptotic proteins including inhibitors of apoptosis (IAPs) have been linked to cancer cell escape from apoptosis [14,15]. A high percentage of pancreatic cancer cell lines and tumors express IAPs, including X-linked IAP (XIAP) [16-18] at elevated levels compared to normal tissue. Manipulating IAPs has been identified as a promising approach for cancer treatment. Second-mitochondria derived activator of caspase (Smac) is a mitochondrial protein released into the cytosol upon apoptosis induction or mitochondrial dysfunction. Smac inhibits IAPs and promotes caspase activation and apoptosis [19,20]. Recently, small-molecule mimetics of Smac have been developed that can promote cancer cell apoptosis either alone or in combination with other proapoptotic agents [16,21,22]. In fact the Smac mimetic JP1201 (JP) has recently been shown to augment the Gem response in PDAC MIA PaCa-2 cells [23].

In the present study we evaluated the effect of JP on the *in vitro *and *in vivo *therapeutic efficacy of various cytotoxic chemotherapy agents in an effort to provide a more effective antitumor strategy for PDAC.

## Methods

### Cell culture and reagents

Human PDAC cell lines, AsPC-1, Panc-1, BxPC-3 and MIA PaCa-2 were obtained from the American Type Culture Collection (ATCC, Rockville, MD). Cell lines were cultured in RPMI 1640 medium (Sigma Chemical Co., St. Louis, MO) supplemented with 10% fetal bovine serum (FBS) and 100 U/ml penicillin/streptomycin solution (Sigma) at 37°C in a humidified 5% CO_2 _atmosphere. JP was obtained from Joyant Pharmaceuticals (Dallas, TX), Gem was purchased from Eli Lilly Corporations (Indianapolis, IN), doxorubicin (Dox) was purchased from Ben Venue Laboratories (Bedford, OH) and docetaxel (DT) was purchased from Sanofi-aventis (Bridgewater, NJ).

### Cell viability assay

*In vitro *cell viability of PDAC cell lines was evaluated by using the colorimetric WST-1 reagent (Roche Diagnostics, Indianapolis, IN) as described earlier [24]. The assay is based on the ability of viable cells to cleave the sulfonated tetrazolium salt WST-1 (4-[3-(4-iodophenyl)-2-(4-nitrophenyl)-2H-5-tetrazolio]-1,3-benzene disulfonate) by mitochondrial dehydrogenases. Briefly, PDAC cells (4,000 cells per well) were plated in a 96-well plate in regular growth medium and after 16 hours the medium was replaced with 2% FBS containing medium. After 5 hours incubation the cells were treated with JP, Gem, Dox or DT, either alone or in combination. After additional incubation of 72 hours, 10 μl WST-1 reagent was added in each well followed by incubation for 2 hours. The absorbance at 450 nm was measured using a microplate reader.

### Western blot analysis

A monolayer of cells at 75 to 80% confluence was placed in 2% FBS containing medium for at least 5 hour before treatment with JP (10 μM), gemcitabine (10 μM) for 24 hours. Cells were lysed and equal amounts of total protein were separated by SDS-PAGE and transferred to PVDF membranes (Bio-Rad, Hercules, CA). The membranes were blocked for 1 hour at room temperature with gentle shaking in TBS-T (10 mM Tris-HCl (pH 7.6), 150 mM NaCl, 0.05% Tween 20). The membranes were incubated overnight at 4°C with the following antibodies: phospho-JNK, (Santa Cruz Biotechnologies, Santa Cruz, CA), poly (ADP-ribose) polymerase-1 (PARP-1) (Cell Signaling Technology, Beverly, MA), α-tubulin or GAPDH (Sigma). The membranes were then incubated with corresponding HRP-conjugated secondary antibodies (Pierce Biotechnologies, Rockford, IL) for 1 hour at room temperature. Specific bands were detected using the enhanced chemiluminescence reagent (ECL, Perkin Elmer Life Sciences, Boston, MA) on autoradiographic film and quantitated by densitometry.

### Apoptosis assay by Annexin V-FITC and propidium iodide (PI) staining

Effect of JP and Gem on AsPC-1 cell apoptosis was detected by using the Annexin V-FITC apoptosis detection kit (BioVision, CA) as per manufacturer's protocol. Briefly, AsPC-1 cells were grown in 10% serum containing medium up to 80% confluence, medium was changed to 2% serum containing medium and incubated for 5 hours and then treated with JP (10 μM) and Gem (10 μM) for 12 hours. After incubation, cells were trypsinized and suspended in 1 ml of low serum medium. From each cell suspension 2 × 10^5 ^cell were centrifuged and re-suspended in 500 μl 1X binding buffer. In each sample 5 μl of Annexin V-FITC and 5 μl of PI was added, incubated for 5 minutes in dark and immediately analyzed by flow cytometry (FACS Calibur System, BD Biosciences, Franklin Lakes, NJ). The annexin V - FITC positive and PI-negative cells were considered to be early apoptotic cells.

### Animal Studies

All animal studies were performed in accordance with the Institutional Animal Care and Use Committee at the University of Texas Southwestern Medical Center (Dallas, TX). *In vivo *animal studies were performed using 4-6 weeks old SCID mice purchased from an on campus facility. Tumor growth experiments were performed by orthotopically injecting 1 × 10^6 ^Panc-1 cells. Animals were examined by ultrasound and randomized into treatment groups when tumor size averaged 500 mm^3 ^at approximately 8 weeks after tumor cell injection. Animals were injected intraperitoneally with PBS (control), JP (6 mg/kg in 100 μl PBS, three times weekly) and Gem (25 mg/kg in 100 μl PBS, three times weekly), either alone or in combination. Three mice from each treatment group were sacrificed at 24 hours of therapy while the remaining animals in each group received 2 weeks of therapy prior to sacrifice.

Animal survival studies were conducted in an intraperitoneal PDAC tumor model as previously described [25]. Female SCID mice received 0.75 × 10^6 ^human AsPC-1 cells intraperitoneally. The animals were randomly grouped (n = 6 to 8 per group) and treated intraperitoneally with PBS (control), JP (6 mg/kg in 100 μl PBS, twice weekly), Gem (100 mg/kg in 100 μl PBS, twice weekly) and DT (3 mg/kg in 100 μl PBS, twice weekly), either alone or in combination for 14 days or as maintenance therapy. Animal weight was measured twice weekly and all animals were examined daily for signs of distress or development of jaundice. Moribund mice at risk for distress were euthanized in accordance with the local animal care committee protocol. Subsequently, animals were examined for presence and extent of intra-abdominal tumor.

### Statistical analysis

*In vitro *cell proliferation assay results are expressed as mean ± standard deviation. Statistical significance was analyzed by the two-tailed Student's t-test using GraphPad Prism 4 Software (GraphPad Software, San Diego, CA). For *in vivo *studies, statistical analysis was performed by ANOVA for multiple group comparison and Student's t-test for the individual group comparison. In survival studies, statistical differences were analyzed by nonparametric survival statistics and logrank test. Values of p < 0.05 were considered to represent statistically significant group difference.

## Results

### Effect of JP and Gem on PDAC cell proliferation

**Figure 1:**
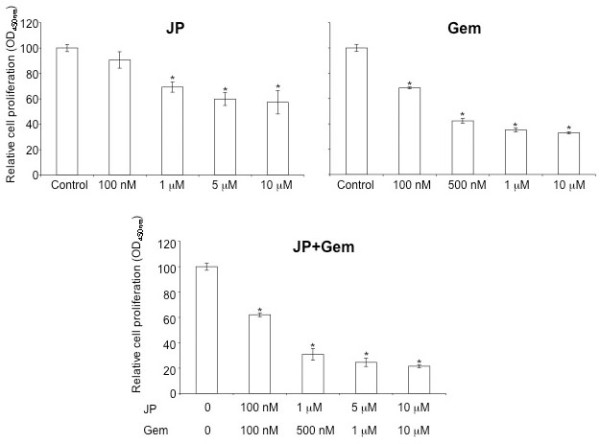
**Effects of JP and Gem on AsPC-1 cell proliferation *in vitro***. AsPC-1 cells were plated on a 96-well plate and were treated with PBS (Control, C), JP or Gem, either alone or in combination. After 72 hours incubation, 10 μl WST-1 reagent was added in each well and incubated for additional 2 hours. The resulting number of viable cells was calculated by measuring absorbance of color produced in each well. Data are representative of mean values ± SD of triplicate determinants. Symbol * represents P values of less than 0.0004.

*In vitro *WST-1 assay was performed to examine the effect of JP and Gem on PDAC cell proliferation. In AsPC-1 cells, JP and Gem significantly inhibited the cell proliferation. After 72 hours of incubation, JP (1 μM) and Gem (500 nM) inhibited the AsPC-1 cell proliferation by 31% and 58%, respectively (Figure 1). The combination of JP and Gem had an additive effect on inhibition of AsPC-1 cell proliferation, with an inhibition in cell proliferation of 70% after 72 hours of incubation (Figure 1). At these concentrations, the inhibition in cell proliferation of other PDAC lines in JP, Gem and JP + Gem groups were 54%, 10% and 79% for Panc-1; 42%, 56% and 77% for BxPC-3; and 9%, 43% and 77% for MIA PaCa-2, respectively.

### Effect of JP and Gem on PDAC apoptosis

**Figure 2:**
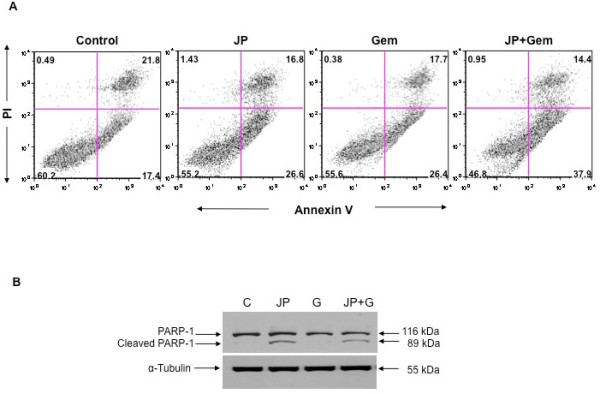
**Effects of JP and Gem on PDAC cell apoptosis**. AsPC-1 cells were treated with JP (10 μM) and Gem (10 μM) either alone or in combination. (A) The early apoptotic cells (lower right quadrant) were measured by flow cytometry after staining with FITC-conjugated annexin V and propidium iodide. The percentage of cells in each quadrant is indicated within the quadrant. (B) PARP-1 cleavage was measured by Western blotting. The total cell lysate was subjected to SDS-PAGE and immunoblotted with PARP-1 antibody, which recognizes full-length (116 kDa) and cleaved (89 kDa) PARP-1 protein. Expression of α-tubulin (55 kDa) was analyzed as loading control on a separate gel. Data are representative of two independent experiments with similar results.

We examined if the inhibition in AsPC-1 cell viability by JP and Gem could in part be due to induction of apoptosis. Annexin V/PI staining assay revealed an increase in early apoptotic cells by JP and Gem treatment that was further increased by combination of these agents (Figure 2A). At 10 μM concentration of either agent, the percentage of early apoptotic cells was 17% in controls, 26% in JP or Gem, and 38% in the JP + Gem group. (Figure 2A).

We also measured cleavage of PARP-1 protein, a caspase-dependent apoptosis-marker protein, after JP and Gem treatment. A significant increase in the expression of cleaved PARP-1 protein was observed after treatment with JP, but not after exposure to Gem (Figure 2B).

### Combination effects of JP and doxorubicin or docetaxel on PDAC cell proliferation

**Figure 3:**
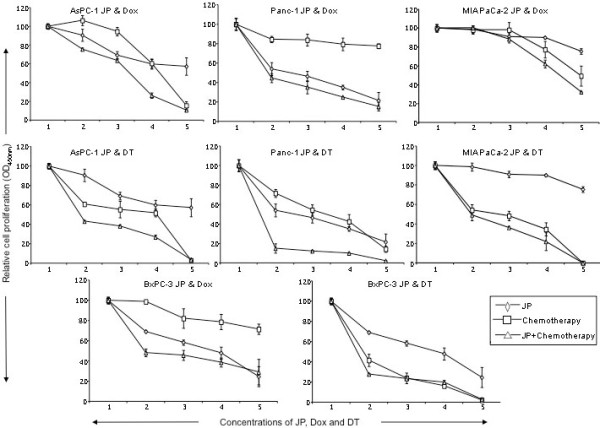
**Effects of JP, doxorubicin and docetaxel on *in vitro *cell proliferation of AsPC-1, Panc-1, MIA PaCa-2 and BxPC-3 PDAC cell lines**. Cells were plated on 96-well plates and treated with JP, Dox or DT, either alone or in combination. X-axis numbers represent treatment concentrations of JP, Dox and DT. Number 1 represents control, number 2 represents JP 100 nM, Dox or DT 10 nM, number 3 represents JP 1 μM, Dox or DT 100 nM, number 4 represents JP 5 μM, Dox or DT 1 μM, and number 5 represents JP 10 μM, Dox or DT 10 μM.

We next evaluated if JP can sensitize other chemotherapeutic agents. *In vitro *WST-1 assay revealed that JP, Dox and DT inhibited the proliferation of all four PDAC cell lines tested in a dose-dependent manner (Figure 3). Interestingly, the combinations of JP with either doxorubicin or docetaxel had additive effects on inhibition of proliferation of PDAC cell lines (Figure 3). At intermediate concentrations of these agents, inhibition in cell proliferation in AsPC-1 cells in JP (5 μM), Dox (1 μM), DT (1 μM), JP + Dox and JP + DT groups were 40%, 39%, 48%, 73% and 73%, respectively. Similar additive combination effects of JP with Dox or DT were observed regarding Panc-1, MIA PaCa-2 and BxPC-3 proliferation (Figure 3).

**Figure 4:**
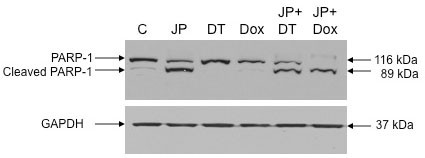
**Effects of JP, Dox and DT on PAPR-1 cleavage**. AsPC-1 cells were treated with JP (10 μM), Dox (10 μM) or DT (10 μM), either alone or in combination. PARP-1 cleavage was measured by Western blotting. The total cell lysate was subjected to SDS-PAGE and immunoblotted with PARP-1 antibody, which recognizes full-length (116 kDa) and cleaved (89 kDa) PARP-1 protein. Expression of GAPDH (37 kDa) was analyzed as loading control on a separate gel. Data are representative of two independent experiments with similar results.

Evaluation of PARP-1 cleavage after JP, Dox and DT treatment by Western blot analysis revealed that Dox and DT had no effect on PARP-1 cleavage, while JP exposure led to a significant increase in PARP-1 cleavage (Figure 4).

### *In vivo *effects of JP addition to gemcitabine and docetaxel

**Figure 5:**
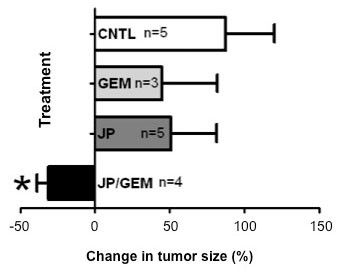
**Effects of JP and Gem on animal tumor burden**. Panc-1 cells (1 × 10^6^) were injected orthotopically in SCID mice. Eight weeks after tumor cell injection mice were randomized and treated with PBS (control), JP, Gem and JP + Gem. Three mice in each group were sacrificed at 24 hours of therapy, with a mean tumor weight of 0.594 ± 0.08 g. The remaining mice were sacrificed after 2 weeks. Symbol * represents p < 0.05 verses control (CNTL).

**Figure 6:**
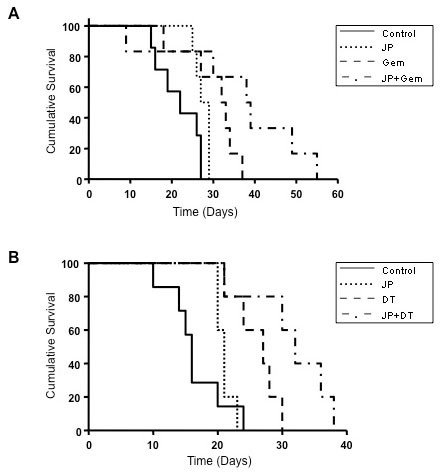
**Effects of JP, Gem and DT, either alone or in combination on animal survival**. AsPC-1 cells (0.75 × 10^6^) were injected intraperitoneally in SCID mice and treatment started after 12 days. (A) Mice were treated with PBS (control), JP, Gem and JP + Gem for 14 days. The curve represents the survival time from the beginning of therapy. The p values for survival differences after Gem and JP + Gem versus control are 0.02 and 0.01, respectively; JP + Gem versus Gem: p = 0.06; JP + Gem versus JP: p = 0.02. (B) Mice were treated with PBS (control), JP, DT and JP + DT for 14 days. The curve represents the survival time from the beginning of therapy. The p values for survival differences after DT or JP + DT treatment versus control were 0.006; JP + Gem versus Gem: p = 0.05; JP + Gem versus JP: p = 0.04.

The antitumor impact of JP, Gem and DT, either alone or in combination, was evaluated in murine PDAC xenografts. In an orthotopic Panc-1 xenograft model, tumor weights were measured at completion of therapy and compared to tumors harvested at 24 hours of therapy (Figure 5). The increase in tumor weight was 87% in controls, while Gem and JP treatment alone trended towards decreased tumor growth, albeit not significant. JP + Gem combination treatment resulted in decreased tumor weight compared to that at treatment start, with a mean reduction of approximately 30% (p < 0.05 verses control) (Figure 5). In survival studies with maintenance therapy, a statistically significant improvement in animal survival was observed in mice treated with the combination of JP + Gem (median: 38 days, p = 0.01) compared to controls (22 days) or single agent treatment with JP (28 days) or Gem (32 days, p = 0.02) (Figure 6A). Animal survival was also significantly improved in a 14-day combination treatment with JP + DT (32 days) as compared to controls (16 days) or single agent treatment with JP (21 days) or DT (27 days) (Figure 6B). In addition to survival impact, we also evaluated the treatment effects of JP and DT on inhibition of local tumor growth in subcutaneous AsPC-1 pancreatic cancer xenografts. JP enhanced the DT-mediated local antitumor effects: compared with controls, addition of JP enhanced the inhibition in net tumor growth by DT of 57% to 91% in combination (p = NS), respectively (data not shown).

## Discussion

Resistance to conventional chemotherapy continues to be a challenge in PDAC. Acquired resistance to apoptosis, which is prevalent in PDAC, is a critical pathway that promotes resistance to conventional chemotherapy [17,18]. Therefore, targeting apoptosis resistance has emerged as an attractive novel cancer therapeutic strategy [26,27]. Small molecules with proapoptotic activity are particularly advantageous, as they are membrane permeable, inhibit antiapoptotic molecules, augment efficacy of current therapeutics and minimize side effects of single agent therapy. Smac mimetics represent a novel class of anticancer drugs that are currently undergoing clinical evaluation [28]. We studied the combination treatment effects of a novel Smac mimetic, JP1201, in combination with the deoxycytidine analogue gemcitabine and the cell-mitosis inhibitor docetaxel in experimental pancreatic cancer.

Human PDAC cells lines have been shown to display marked heterogeneity towards Gem [29]. A similar heterogeneity regarding Gem sensitivity was seen in our four lines tested. Nevertheless, we observed that JP inhibited the proliferation of all four PDAC cell lines, and that the combination of JP + Gem had additive effects. Antitumor activity of Smac mimetics is mediated through induction of apoptosis. We therefore explored if proliferation inhibition of PDAC cells after combination therapy is in part due to induction in apoptosis. Detection of early apoptotic cells by annexin V/PI staining demonstrated that JP and Gem moderately induced apoptosis, and JP + Gem had an additive effect. Smac mimetic-induced apoptosis involves caspase activation that cleaves PARP-1, a DNA repair enzyme, to produce 89 kDa or 24 kDa cleavage product. We observed a dramatic increase in the 89 kDa C-terminus cleavage product of PARP-1 after JP treatment indicating the involvement of caspases in JP-induced apoptosis. While Gem treatment for 12 hours caused an increase in annexin V positive cells, no PARP-1 cleavage was detected after 24 hours of Gem treatment; this finding is likely related to the fact that 24 hours incubation may not be enough to cause detectable levels of PARP-1 cleavage. Based on *in vitro *additive anti-proliferative and proapoptotic effects of JP and Gem together, we examined effects of these agents on *in vivo *animal survival and observed that the JP + Gem combination significantly increases the animal survival compared with controls or monotherapy. Our findings corroborate a recently published report [23] that the Smac mimetic JP1201 enhances chemotherapy response of Gem in PDAC cell lines; accordingly, the JP-mediated increase in antiproliferative response after Gem was greatest in Panc-1 cells followed by MIA PaCa-2, BxPC-3 and AsPC-1 cells. Correspondingly, a 30% reduction in tumor weight was observed in our orthotopic Panc-1 tumor experiment compared to a 50% reduction in orthotopic MIA PaCa-2 tumors in the previous study [23]. In addition, a significant improvement in animal survival was observed with JP + Gem treatment in AsPC-1 xenografts compared with JP or Gem alone similar to the previous study [23]. Of note, the lack of a significant reduction in tumor growth (Figure 5) by Gem alone in the present study is likely merely a result of the small number of animals in that tumor growth experiment, and does not pose a conflict with the significant improvement in animal survival seen after the same treatment (Figure 6A). Altogether, our results can thus support a more generalizable phenomenon in this context, as JP combination benefits have been obtained in four PDAC cell lines, and with other cytotoxic agents beyond gemcitabine.

Smac mimetics have been shown to enhance antitumor effects of several agents including cisplatin [30] and TRAIL [23,31] in different cancer types. Docetaxel is a clinically well established anti-mitotic chemotherapy treatment for several cancers including breast, ovarian and non-small cell lung cancer [32]. We explored the combination treatment effects of JP with other chemotherapy agents such as doxorubicin and docetaxel in experimental pancreatic cancer. *In vitro *studies showed that JP significantly enhanced antiproliferative effects of Dox and DT in all four PDAC cell lines tested. In addition JP and DT combination had significant enhanced effect on tumor regression and animal survival in pancreatic cancer xenografts. These results indicate that a potential clinical benefit to Smac-mimetic combination therapies does not appear to be chemotherapeutic agent specific, and that such approach may carry a wide range of indications.

Small molecule Smac mimetics have been shown to be particularly advantageous in overcoming chemotherapy resistance when resistance occurs through modulation of the NFkB-IAP pathway. Since several pancreatic cancer cell lines and tumors have been shown to overexpress IAPs (16-18), treatment of PDAC with JP in combination with other chemotherapeutic agents shows specific promise for becoming effective for this disease. Our present study supports this notion through preliminary, preclinical evidence. The potential to render traditionally non-effective agents more effective for clinical PDAC therapy is particularly intriguing in this context.

## Conclusions

Chemotherapy-induced apoptosis of PDAC can be enhanced through JP1201, a Smac mimetic. The resulting combination improves apoptotic response, antiproliferative effects, local tumor control, and animal survival. This strategy shows promise for future clinical evaluation.

## Abbreviations

PDAC: pancreatic ductal adenocarcinoma; Gem or G: gemcitabine; DT: docetaxel; PARP-1: poly (ADP-ribose) polymerase-1; WST-1: 4-[3-(4-iodophenyl)-2-(4-nitrophenyl)-2H-5-tetrazolio]-1,3-benzene disulfonate; EC: endothelial cell.

## Competing interests

The authors declare that they have no competing interests.

## Authors' contributions

NA was involved in the design of the study, execution of the experiments, data analysis and drafting the manuscript. AK and JET performed the orthotopic tumor growth studies. MAS participated in the animal survival studies. RAB contributed to the coordination of the study and manuscript preparation. RES conceived of the study, and was involved in the planning and design of the study, data analysis and drafting of the manuscript. All the authors read and approved the manuscript.

## Pre-publication history

The pre-publication history for this paper can be accessed here:

http://www.biomedcentral.com/1471-2407/11/15/prepub
